# Heterogeneity of Bovine Peripheral Blood Monocytes

**DOI:** 10.3389/fimmu.2017.01875

**Published:** 2017-12-19

**Authors:** Jamal Hussen, Hans-Joachim Schuberth

**Affiliations:** ^1^Department of Microbiology and Parasitology, College of Veterinary Medicine, King Faisal University, Al Ahsa, Hofuf, Saudi Arabia; ^2^Immunology Unit, University of Veterinary Medicine, Hannover, Germany

**Keywords:** monocyte subsets, monocyte heterogeneity, bovine, macrophages, monocyte migration

## Abstract

Peripheral blood monocytes of several species can be divided into different subpopulations with distinct phenotypic and functional properties. Herein, we aim at reviewing published work regarding the heterogeneity of the recently characterized bovine monocyte subsets. As the heterogeneity of human blood monocytes was widely studied and reviewed, this work focuses on comparing bovine monocyte subsets with their human counterparts regarding their phenotype, adhesion and migration properties, inflammatory and antimicrobial functions, and their ability to interact with neutrophilic granulocytes. In addition, the differentiation of monocyte subsets into functionally polarized macrophages is discussed. Regarding phenotype and distribution in blood, bovine monocyte subsets share similarities with their human counterparts. However, many functional differences exist between monocyte subsets from the two species. In contrast to their pro-inflammatory functions in human, bovine non-classical monocytes show the lowest phagocytosis and reactive oxygen species generation capacity, an absent ability to produce the pro-inflammatory cytokine IL-1β after inflammasome activation, and do not have a role in the early recruitment of neutrophils into inflamed tissues. Classical and intermediate monocytes of both species also differ in their response toward major monocyte-attracting chemokines (CCL2 and CCL5) and neutrophil degranulation products (DGP) *in vitro*. Such differences between homologous monocyte subsets also extend to the development of monocyte-derived macrophages under the influence of chemokines like CCL5 and neutrophil DGP. Whereas the latter induce the differentiation of M1-polarized macrophages in human, bovine monocyte-derived macrophages develop a mixed M1/M2 macrophage phenotype. Although only a few bovine clinical trials analyzed the correlation between changes in monocyte composition and disease, they suggest that functional differences between human and bovine monocyte subsets are also reflected in their different clinical relevance for distinct diseases. In opposite to the human system, where higher blood cell number of non-classical monocytes was widely correlated with several human infectious and non-infectious diseases, higher counts of bovine intermediate monocytes are suggested as a potential biomarker for inflammatory responses postpartum.

## Introduction

Monocytes are bone marrow-derived myeloid cells with central role in immunity to infection or injury (1, 2). In addition to their importance as precursors for tissue macrophages and dendritic cells, monocytes are key member of the innate immune system, with important effector functions during different phases of inflammation (3). They are functionally characterized by their ability to sense pathogens, to phagocytose microbes, to produce cytokines and chemokines, and to present antigens to T cells (4).

For the whole monocyte population, phenotypic and functional properties were intensively investigated in human and murine as well as in different veterinary species. This includes earlier works on bovine (5–7), ovine (8, 9), caprine (10, 11), equine (12, 13), and porcine (14, 15) monocytes and monocyte-derived cells.

For a long time, monocytes were considered as a homogenous population of circulating blood cells. In 1989, Passlick et al. identified distinct human monocyte subsets (16). Based on the differential expression of CD14, the lipopolysaccharide (LPS) receptor and CD16, the FcγIIIR, two subpopulations of human monocytes (CD14^++^ CD16^−^ and CD14^+^ CD16^+^) were initially defined (16). Subsequently, differences within the CD16-positive monocyte fraction enabled the determination of human CD14^++^ CD16^+^ and CD14^+^ CD16^++^ monocyte subsets (17). According to the newly accepted nomenclature of leukocytes, human blood monocytes are currently divided into three different subpopulations based on their CD14 and CD16 expression. The main fraction of human blood monocytes (90%) with the highest CD14 expression but with no CD16 expression (CD14^++^ CD16^−^) are now termed classical monocytes (cM), whereas the minor fraction (10%) contains intermediate monocytes (intM) with high CD14 and low CD16 expression (CD14^++^ CD16^+^), and non-classical monocytes (ncM) with low CD14 and high CD16 expression (CD14^+^ CD16^++^) (18).

Recently, three monocyte subsets have been identified in the bovine peripheral blood (19–22). As bovine monocyte subsets displayed distinct functional differences from their human counterparts (21, 22), this work focuses on the phenotypic and functional characterization of bovine blood monocyte subsets from a comparative point of view.

## Phenotypic Heterogeneity of Bovine Monocyte Subsets

Similar to porcine (23, 24) and rat monocytes (25), the surface protein CD172a, also known as signal-regulatory protein alpha, was defined as a pan marker for bovine monocytes (21). This is in contrast to human and murine monocytes (1), where CD115, the colony-stimulating factor-1 receptor, is used to identify the total monocytes population. Although gene expression analysis indicates the expression of CD115 in bovine monocytes (19), no specific antibodies are currently available for the detection of bovine CD115 protein molecule.

**Figure 1:**
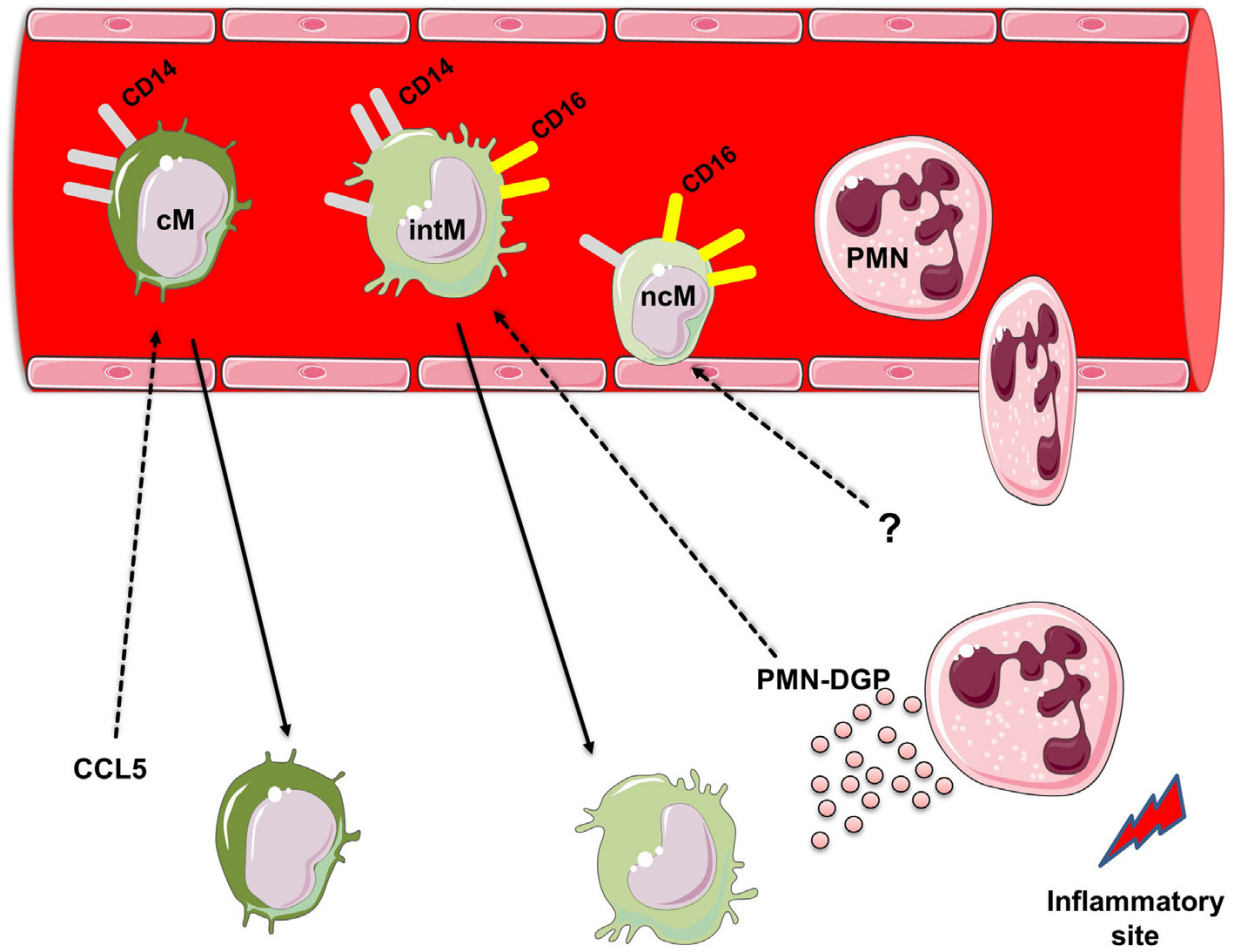
Heterogeneity of bovine monocyte subsets. Bovine monocytes can be subdivided according to their cell surface expression of CD14 and CD16 into three monocyte subsets: (1) bovine classical monocytes (cM) with high CD14 but no CD16 expression (CD14^++^ CD16^−^), (2) bovine intermediate monocytes (intM) with high CD14 and low CD16 expression (CD14^++^ CD16^+^), and (3) bovine non-classical monocytes (ncM) with high CD16 but no CD14 expression (CD14^−^ CD16^++^). Responsiveness of bovine monocyte subsets to the chemokine CCL5 and degranulation products (DGP) of Polymorphonuclear neutrophils (PMN). PMN are the first leukocyte subset recruited to sites of inflammation. During their migration to inflammatory sites PMN release the contents of their granules. PMN-DGP selectively support the adhesion of bovine intM and stimulate their subsequent migration. The chemokine CCL5 induces the activation and migration of bovine cM. Selective chemotactic factors for bovine ncM are not determined yet. For figure design, some elements of the *Servier Medical Art Powerpoint-image-bank* were used (http://www.servier.com).

Similar to human monocytes, bovine monocytes can be subdivided according to the surface expression of CD14 and CD16 into three monocyte subsets (21): (1) bovine cM with high CD14 but no CD16 expression (CD14^++^ CD16^−^), (2) bovine intM with high CD14 and low CD16 expression (CD14^++^ CD16^+^), and (3) bovine ncM with high CD16 but no CD14 expression (CD14^−^ CD16^++^) (Figure 1).

As in human blood (26), bovine cM constitute the majority (89%) of monocytes in blood, whereas bovine intM and ncM present just minor proportions (5–10% for each subset) of total bovine monocytes (21). Phenotypically, bovine monocyte subsets differ in their cell surface expression of different myeloid markers (21). While CD163 is highest expressed on cM, the expression level of CD172a is higher on intM and ncM. IntM are characterized by the highest expression of MHC class II molecules in comparison to the other two subsets (19–21). The surface expression densities of the monocytic markers CD14, CD16, CD163, and MHC-II on human and bovine monocyte subsets (27) indicate a similar immunophenotype of monocyte subsets in the two species (Table 1). This seems also to be the case for the size and granularity of bovine (21) and human (1) ncM, which is smaller and less granular than cM and intM. Therefore, regarding size, immunophenotype and distribution in blood, bovine monocyte subsets seem to share many similarities with their counterparts in human (28).

**Table 1:** Phenotypic profiles of bovine and human monocyte subsets.

	Bovine^a^^,^^b^^,^^c^^,^^d^^,^^e^			Human^f^^,^^g^^,^^h^^,^^i^^,^^j^		
	Classical	Intermediate	Non-classical	Classical	Intermediate	Non-classical
		
	CD14^++^ CD16^-^	CD14^++^ CD16^+^	CD14^+^ CD16^++^	CD14^++^ CD16^-^	CD14^++^ CD16^+^	CD14^+^ CD16^++^
MHC-II	++	+++	+	++	+++	+
CD163	+++	++	−	++	+	−
CD172a	++	++	+++	++	++	+++
CD62L	+++	++	+	+++	++	+
CD11a	+	++	+++	+	++	+++
CD11b	+++	++	+	++	+++	+
CD18	++	+++	+	++	+++	+
CD31	+	+++	+	++	+++	+
CD49d	+	++	+++	+	+	+++
CC chemokine receptor (CCR2)^k^	+++	+++	+	+++	+++	+
CX3CR1^k^	+	++	+++	+	++	+++
CCR5	ND	ND	ND	+	+++	+
Glut 1^l^	+	+	+	±	+	++
Glut 3^l^	+++	++	+	ND	ND	ND

*^a^Hussen et al. (21)*.

*^b^Hussen et al. (29)*.

*^c^Hussen et al. (22)*.

*^d^Corripio-Miyar et al. (19)*.

*^e^Eger et al. (30)*.

*^f^Stansfield and Ingram (31)*,.

*^g^Wong et al. (27)*.

*^h^Zawada et al. (32)*.

*^i^Rogacev et al. (33)*.

*^j^Palmer et al. (34)*.

*^k^Based on gene expression analysis in bovine monocytes (19)*.

*^l^Based on gene expression analysis in bovine monocytes (30)*.

Phenotypic heterogeneity of bovine monocytes is also reflected by expression patterns for a variety of adhesion molecules (Table 1). Similar to human and murine cM (35–37), bovine cM show the highest expression of l-selectin (CD62L) and Mac1 (CD11b/CD18) when compared to intM and ncM (21). A special migratory behavior was described for murine and human ncM, being able to crawl along vascular endothelial cells and to migrate rapidly into tissues upon infection or injury (38, 39). This patrolling behavior is mediated by the integrin LFA1 (CD11a/CD18) (38, 39). In addition, the very late antigen-4 (VLA-4 or CD49d), which promotes monocyte adherence to endothelial vascular cell adhesion molecule-1, has been shown to be highly expressed on human ncM (39, 40). Although it has not been proven by cell adhesion studies, the highest expression level of LFA1 and VLA-4 on bovine ncM suggests a similar patrolling function of this subset in the bovine system (21). Differently from the pattern seen in human and mouse, where ncM have been shown to express the highest level of the platelet endothelial cell adhesion molecule 1 (PECAM1, CD31) (32, 35), the highest level of PECAM1 was found on bovine intM. This may indicate that this molecule primarily contributes to the migration of bovine intM rather than cM or ncM (21).

## Functional Heterogeneity of Bovine Monocyte Subsets

Monocytes are effector immune cells that play key roles during infection or injury. They can phagocytose and kill microbes and produce different cytokines and chemokines (1). The phenotypic heterogeneity of bovine monocyte subsets also extends to their functional properties. Bovine cM have the highest ability to phagocytose bacteria, which is in line with published data to their role in human (32, 39). Bovine intM display an intermediate capacity for phagocytosis, whereas production of reactive oxygen species (ROS) and the gene expression levels for inflammatory cytokines (TNF-α, IL-1β) are clearly strongest in this subset. Conversely, bovine ncM show the lowest phagocytosis and ROS generation capacity. In addition, inflammasome activation in bovine monocytes after combined stimulation with LPS and adenosine triphosphate (21) revealed the nearly absent ability of ncM to produce the pro-inflammatory cytokine IL-1β.

Overall, inflammatory responses to bacterial stimulation, including phagocytosis, ROS generation, and cytokine production, are likely mediated by the CD14-positive fraction of bovine monocytes including cM and intM. This is different from the human system, where the CD16-positive fraction of human monocytes including the intM and ncM was generally termed “pro-inflammatory monocytes” (41, 42). Human CD16-positive monocytes are the main producer of pro-inflammatory cytokines (43, 44) with human ncM producing the highest amount of IL-1β in response to LPS stimulation (27, 45). However, a recent study analyzed the two CD16-positive monocytes separately as intM and ncM and reported a lower ability of human ncM to produce IL-1β in comparison to the other two subsets (46).

In response to infection or injury, a special role was described for murine and human ncM in the early recruitment of neutrophils into inflamed tissues (38). In the bovine system, the low mRNA expression of *CXCL1* and *CXCL8*, genes encoding for two important neutrophil chemokines, in bovine ncM, which could not be upregulated after stimulation with LPS, suggests a marginal role of bovine ncM in the early migration of neutrophils into the inflamed tissue (21).

## Responsiveness to Selected Bovine Chemokines

Monocytes, after their production in the bone marrow, are released into the blood stream, where they circulate for several days before entering tissues (1, 47, 48). The extravasation of monocytes into tissues, which is an important step for effective control and clearance of pathogens (49), includes serial interactions between monocytes, endothelial cells, and chemoattracting factors (50). For the interaction with chemokines, monocytes are equipped with a multitude of chemokine receptors like CC chemokine receptor (CCR) 1, CCR2, CCR5, CXCR4, CCR7, CCR8, CXCR1, CXCR2, and CX_3_C-chemokine receptor 1 (51–56). Recent human and mouse studies have indicated the involvement of distinct chemokine receptors for the migration of different monocyte subsets (50). Human and murine cM express the highest level of CCR2, suggesting a preferential role for CC chemokine (CCL) 2 in their migration (57, 58). Conversely, the highest expression level of CX_3_CR1, the receptor for the chemokine fractalkine (CX_3_CL1), was found on human and murine ncM (17, 38, 39). Although, a similar expression pattern of CCR2 and CX_3_CR1 has been recently reported for bovine monocyte subsets (19), the functional analysis using bovine and human chemokines revealed different responses of monocytes to chemokines in the two species. In contrast to human cM, which have high responsiveness to CCL2 (57, 58), bovine cM are neither activated by nor migrate toward CCL2 (22). As these data are merely based on *in vitro* studies with sorted blood monocytes, a different mode of action for bovine CCL2 *in vivo* cannot be excluded (50). On the other hand, CCL5 selectively activates bovine cM and induces their migration *in vitro* (Figure 1). This is also different from the human system, where human intM express the highest level of CCR5 (17, 33). The absent responsiveness of bovine ncM toward the chemokines CCL2 and CCL5 seems to be paralleled by findings in human, where ncM have been shown to lack the expression of CCR2, but express the highest level of Cx_3_CR_1_ and were therefore selectively attracted by CX_3_CL_1_ (59). However, functional studies on the responsiveness of bovine monocyte subsets toward other monocytic chemokines such as CX_3_CL_1_ are still to be done. Such studies would pave the way for the development of prophylactic and therapeutic approaches aiming at the selective modulation (enhancement or inhibition) of the migratory properties of distinct monocyte subsets.

## Responsiveness toward Neutrophil Degranulation Products (DGP)

Polymorphonuclear neutrophils (PMN) are important elements of the innate immune response and represent an essential cooperation partner of monocytes during all phases of inflammation (60). The acute phase of an inflammatory response is characterized by an early extravasation of neutrophils to the inflamed site (61, 62). These early recruited neutrophils are believed to contribute to the recruitment of blood monocytes by several mechanisms including the release of neutrophil granule proteins (60, 63, 64). Recent studies in human (65) and mice (66) have provided evidence for the importance of PMN DGP in the interaction with distinct monocyte subsets (60). Also, in the bovine system monocyte subsets show a heterogenic responsiveness toward PMN-DGP (29). As measured by their Ca^2+^-influx-inducing potential, DGP of bovine neutrophils induce a selective activation of bovine cM and intM. This is in line with findings in the murine system, where stimulation with PMN-DGP results in Ca^2+^-mobilization in inflammatory monocytes, which include both the murine cM and intM (66). However, a selective migration-inducing potential of PMN-DGP was only demonstrated for bovine intM (Figure 1). This is also supported by the selective upregulation of the adhesion molecules CD31 and CD11a on bovine intM stimulated with PMN-DGP (29). The lack of a responsiveness of bovine ncM toward PMN-DGP is paralleled by findings in the human system (18).

## Developmental Relationship Between Bovine Monocyte Subsets

Some studies suggested a developmental relationship between the three monocyte subsets, with intM representing transitional cells bridging cM and ncM. According to this hypothesis, monocytes leave the bone marrow as cM, which can differentiate into intM and further into ncM in peripheral blood (18, 67, 68). The *in vitro* stimulation with the T-helper 1 cytokine IFNγ induces the upregulation of CD16 on bovine cM which results in an increased fraction of intM but does not induce a shift from intM into ncM. This effect seems to be selective for IFNγ, as the stimulation with T-helper-2 cytokines (IL-4 and IL-13), pro-inflammatory cytokines (IL-1β or TNF-α), or the chemokine CCL5 did not induce any change in the distribution of bovine monocyte subsets (21). Partially in line with this, the treatment of patients with a combination of IFNγ and M-CSF enhanced CD16 expression on human cM and the expansion of CD16-positive monocytes (68). However, studies are needed to analyze the potential of sorted human, murine, or bovine cM to develop into intM and further into ncM.

## Macrophages Derived from Monocyte Subsets

After leaving the blood stream, monocytes differentiate into tissue monocyte-derived macrophages or dendritic cells (69–73). Gene expression profiles of macrophages generated *in vitro* from bovine monocyte subsets indicate a heterogenic differentiation potential of bovine monocyte subsets into distinct macrophage subtypes (74). The highest expression of *TNF, IL1, NOS2*, and *CXCL8* in LPS-stimulated macrophages derived from bovine cM and intM argues for a more inflammatory phenotype for cM- and intM-derived macrophages. On the other hand, the highest expression of *ARG1* in ncM-derived macrophages indicates a more anti-inflammatory phenotype for ncM-derived macrophages (74).

**Figure 2:**
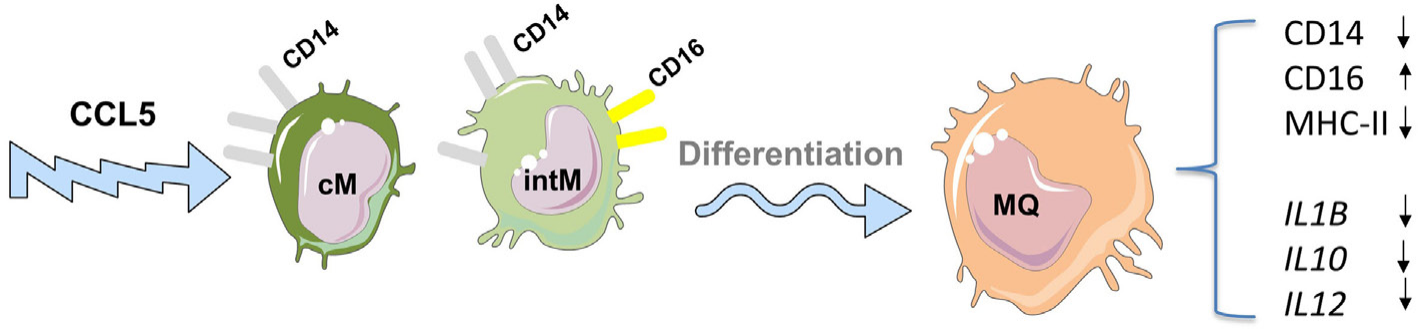
Impact of the chemokine CCL5 on the differentiation of bovine monocyte subsets into macrophages. CCL5 guides the differentiation of bovine CD14-positive monocytes into macrophages with increased expression of CD16 but reduced expression of CD14 and MHC-II molecules. CCL5-differentiated macrophages show reduced responsiveness toward lipopolysaccharide stimulation including reduced expression of genes associated with M1 as well as M2 macrophages indicating the development of an endotoxin-tolerance-similar status (ET) in CCL5-differentiated macrophages rather than a polarized macrophage phenotype. For figure design, some elements of the *Servier Medical Art Powerpoint-image-bank* were used (http://www.servier.com).

The differentiation of monocytes into macrophages can also be guided by different local mediators like cytokines, chemokines, and microbial products resulting in different functional macrophage subtypes (75–78). The chemokine CCL5 has been shown to guide the differentiation of bovine CD14-positive monocytes into macrophages with increased CD16 expression but reduced expression of CD14 and MHC-II molecules (22). The analysis of gene expression revealed a reduced responsiveness of CCL5-differentiated macrophages toward LPS stimulation, as seen in the reduced expression of M1 (*IL6, CXCL8*) as well as M2 (*IL10* and *ARG1*) macrophage genes (Figure 2). This indicates the development of an endotoxin-tolerance-similar status (ET) in CCL5-differentiated macrophages rather than a polarized macrophage phenotype (22).

**Figure 3:**
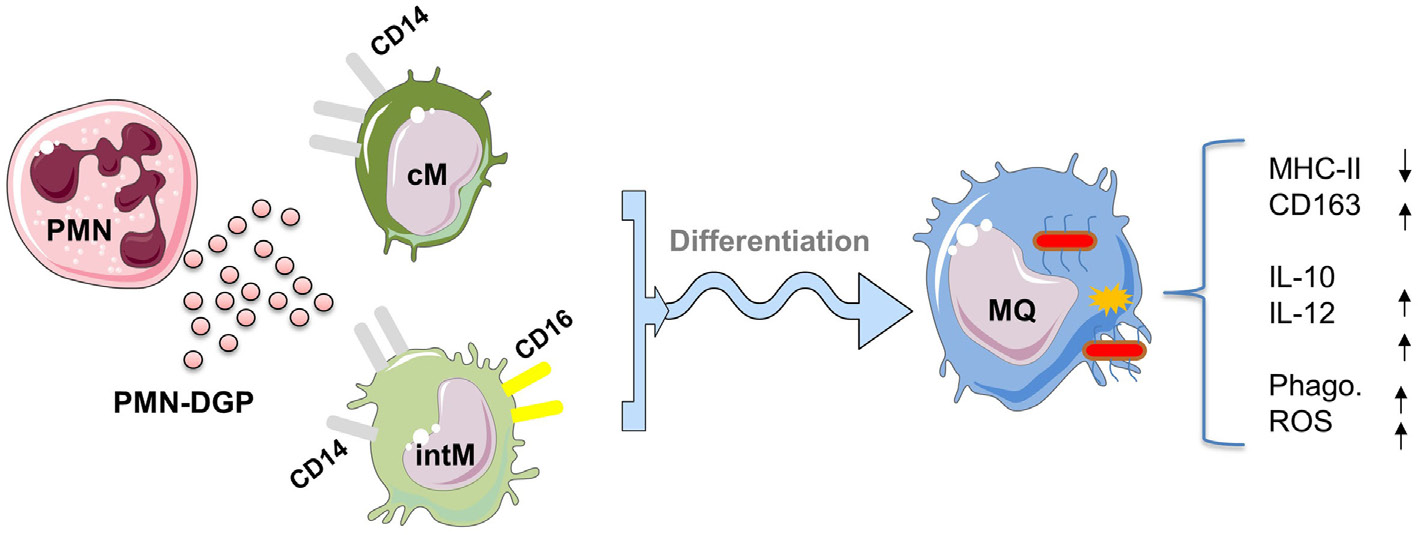
Impact of degranulation products (DGP) of Polymorphonuclear neutrophils (PMN) on the differentiation of CD14-positive monocytes into macrophages. PMN-DGP, when present during the *in vitro* differentiation of bovine classical (cM) or intermediate monocytes (intM), shape the phenotype and function of monocyte-derived macrophages. Monocyte-derived macrophages developed under PMN-DGP display phenotypic features of M2 macrophages (reduced expression of MHC class II molecules and enhanced expression of CD163). Functionally, these macrophages show increased production of the anti-inflammatory cytokine IL-10 and the inflammatory cytokine IL-12 with enhanced antimicrobial activities (phagocytosis, ROS production). This indicates that PMN-DGP guide the differentiation of bovine CD14-positive monocytes (cM and intM) toward a mixed macrophage phenotype with enhanced antimicrobial functions. For figure design, some elements of the *Servier Medical Art Powerpoint-image-bank* were used (http://www.servier.com).

Also DGP of neutrophils, when present during the *in vitro* differentiation of bovine cM and intM, shape the phenotype and function of monocyte-derived macrophages (29). Although, monocyte-derived macrophages developed under PMN-DGP display features of M2 macrophages (reduced expression of MHC class II molecules and enhanced expression of CD163) (79), the function of these cells (increased production of the anti-inflammatory cytokine IL-10 as well as the pro-inflammatory cytokine IL-12) argues against a strong polarizing effect of PMN-DGP (Figure 3). This is in contrast to the human system, where cathelicidin LL-37, a DGP of human neutrophils, induced the differentiation of M1 monocyte-derived macrophages (80). The mixed M1/M2 phenotype of bovine monocyte-derived macrophages may depend on a species-specific composition of PMN granule molecules which has been shown to differ significantly between human and bovine neutrophils (81). However, the impact of PMN-DGP on the antimicrobial activity (phagocytosis, ROS production) of human (82) and bovine monocyte-derived macrophages (29) is comparable. Collectively, PMN-DGP guide the differentiation of bovine CD14-positive monocytes toward a mixed macrophage phenotype with enhanced antimicrobial functions (29).

## Clinical Relevance of Bovine Monocyte Subsets

In human, increased percentages of CD16-positive monocyte fraction were reported in several infectious and non-infectious diseases (31, 83). Especially in patients with severe bacterial sepsis or tuberculosis, increased percentages of human CD16-positive monocytes correlated with disease severity (42, 84, 85).

The functional heterogeneity of bovine monocyte subsets suggests that these subsets may be of clinical relevance for distinct bovine diseases, especially in the postpartal period of dairy cows with high incidences of infectious diseases like mastitis and endometritis (86).

The period around parturition is characterized by changes in the number of circulating monocytes (30, 87, 88) with maximum blood cell counts for all three bovine monocyte subsets at day 7 after parturition. This increase in monocyte numbers was correlated with hormonal changes and changes in milk production around parturition (36). Although blood cell counts of all three monocyte subsets are higher in cows with postpartal mastitis or metritis in comparison to healthy cows, the increase was especially pronounced for the two CD16-positive monocyte subsets intM and ncM (30). Whether this increase in intM and ncM relies on an enhanced production of these subsets in the bone marrow or whether it is the result of reduced adhesion to endothelial cells is still unknown.

The relationship between prepartum cell composition of bovine monocyte subsets and the occurrence of mastitis or endometritis postpartum has been recently analyzed (89). The study has shown that the composition of monocyte subsets before calving is in relation to the susceptibility of cows to infectious diseases within 2 weeks postpartum. Higher counts of circulating CD14-negative monocytes prior to calving reduced the probability of postpartal infectious mastitis and/or endometritis, whereas an increase in CD14-positive monocyte counts prior to calving increased the susceptibility to infectious diseases postpartum.

In another clinical study, the changes in peripheral blood monocytes associated with bovine subclinical endometritis were evaluated (90). Although the cell numbers of all three monocyte subsets were higher in cows with subclinical endometritis, no selective increase in either of monocyte subsets was observed. However, selectively intM have been shown to be responsible for the enhanced expression of inflammatory gens in leukocytes of diseased animals. Plasma from diseased animals induced an elevated expression of genes encoding for the inflammatory mediators *CXCL8, CXCL1*, and *IL1B* in intM (90). The clinical relevance of bovine intM as a pro-inflammatory monocyte with an important role during the acute phase of inflammation has been confirmed in a recent work on calves with adjuvant-induced skin inflammation (91). The study has demonstrated a potent recruitment of bovine intM to the draining lymph node after the onset of a local skin inflammation with upregulated genes for pro-inflammatory cytokines.

During the postpartal period, negative energy balance, especially in high-yielding dairy cows, is often associated with limited energy supply for immune cells and therefore with altered peripartal immune responsiveness (87, 92, 93). In a recent study, glucose uptake capacities of bovine monocyte subsets during the peripartal period were evaluated (30). In cows, unstimulated monocyte subsets cM and intM take up more glucose compared to ncM. This is in contrast to the human system, where glucose uptake capacity does not differ among unstimulated human monocyte subsets (34). As bovine cM and intM display the highest phagocytosis and ROS production capabilities when compared to ncM, the higher glucose uptake capacities of these subsets might therefore be in line with their functional activities (21).

## Conclusion and Perspectives

In the bovine peripheral blood, the expression of the cell-surface molecules CD14 and CD16 defines bovine classical (CD14^++^ CD16^−^), intermediate (CD14^++^ CD16^+^), and non-classical (CD14^−^ CD16^++^) monocyte subsets. Regarding their distribution in blood and phenotype, bovine monocyte subsets share similarities with human monocyte subsets. However, many functional differences exist between monocyte subsets from the two species. Although the studies discussed above provide basic knowledge about the heterogeneity of bovine monocyte subsets, further studies are needed for the detailed functional analysis of these subsets. Especially, the mechanisms that control the development of bone marrow precursor cells into distinct monocyte subsets remain to be investigated. Although bovine classical and intM subsets have been shown to be essentially responsible for the anti-microbial and pro-inflammatory responses, the function of bovine ncM is still obscure. In addition, while bovine cM are selectively attracted by CCL5 and bovine intM by neutrophils DGP, chemotactic factors responsible for a selective recruitment of bovine ncM remain to be determined. Due to their importance as one of the links between innate and adaptive immunity and given the progress that has been recently made in the characterization of dendritic cell subsets in different veterinary species (14), it would also be interesting to investigate the potential of bovine monocyte subsets to differentiate into distinct subsets of dendritic cells. Furthermore, more studies are needed to clarify the clinical relevance of the three subsets in different bovine diseases. It is especially unclear whether higher CD16-positive monocyte cell numbers in diseased animals represent a beneficial or a critical factor.

## Author Contributions

JH and H-JS prepared the figures and tables and wrote the manuscript.

## Conflict of Interest Statement

The authors declare that the research was conducted in the absence of any commercial or financial relationships that could be construed as a potential conflict of interest.
